# A DNA algorithm for the job shop scheduling problem based on the Adleman-Lipton model

**DOI:** 10.1371/journal.pone.0242083

**Published:** 2020-12-02

**Authors:** Xiang Tian, Xiyu Liu, Hongyan Zhang, Minghe Sun, Yuzhen Zhao

**Affiliations:** 1 Business School, Shandong Normal University, Jinan, China; 2 College of Business, The University of Texas at San Antonio, San Antonio, TX, United States of America; Chang Gung University, TAIWAN

## Abstract

A DNA (DeoxyriboNucleic Acid) algorithm is proposed to solve the job shop scheduling problem. An encoding scheme for the problem is developed and DNA computing operations are proposed for the algorithm. After an initial solution is constructed, all possible solutions are generated. DNA computing operations are then used to find an optimal schedule. The DNA algorithm is proved to have an *O*(*n*^2^) complexity and the length of the final strand of the optimal schedule is within appropriate range. Experiment with 58 benchmark instances show that the proposed DNA algorithm outperforms other comparative heuristics.

## 1. Introduction

It is well known that the traditional silicon-based computers use serial algorithms, so that their computing speed cannot qualitatively leap. It is also well known that optimal solutions of most of the celebrated computationally intractable problems can only be found by an exhaustive search through all possible solutions. However, the insurmountable difficulty lies in the fact that such an exhaustive search is too vast to carry out using currently available computing technology, so that numerous intractable problems cannot be solved effectively. Some visionary remarks were made about new ways of solving such problems through possible miniaturizations. Feynman’s view [1] was widely accepted, stating the possibility of establishing “sub-microscopic” computers. Since then, although significant progresses have been made in relation to computer miniaturization, the goal of sub-microscopic computers has not yet been achieved.

As a new interdisciplinary area, DNA computing has received increasing attentions. Massive parallelism and huge storage capacity are two significant advantages of DNA computing. Parallelism means DNA computing can perform billions of operations simultaneously. Furthermore, DNA computers can solve more intractable problems, such as non-deterministic polynomial-time) (NP)-hard problems, in linear time, as compared with conventional electronic computers in exponential time. In addition, the high density of data stored in DNA strands and the ease in duplicating them can make such exhaustive searches possible. Adleman’s experiment [2] solved the Hamiltonian Path Problem for a given directed graph, and demonstrated the strong parallel computing power of DNA computing. Lipton’s DNA-based solution of the satisfiability problem [3] used some of Adleman’s basic operations. Indeed, it used an exhaustive search that was made computationally feasible by the massive parallelism of the DNA strands. Ouyang et al. [4] turned the maximal clique problem, another NP-complete problem, into the maximum independent set problem, and solved the problem with six vertices in the laboratory by using the parallel overlap assembly technology. Roweis et al. [5] introduced a new DNA computing model, i.e., the sticker model, and used this model to solve the minimal set cover problem and the data encryption problem. Furthermore, the self-assembly model [6], the hairpin model [7] and the surface-based model [8, 9] had already been proposed and built.

Among the many DNA computing models mentioned above, the Adleman-Lipton model and the sticker model are most widely used in solving classical combinatorial optimization problems. There are numerous publications in the literature addressing the combinatorial optimization problems using the Adleman-Lipton model. For example, Xiao et al. [10] solved maximum cut problems using the Adleman-Lipton model with *O*(*n*^2^) steps. Hsieh et al. [11] solved the graph isomorphism problem with the Adleman-Lipton model with stickers using a polynomial number of basic biological operations. Yang et al. [12] proposed a theoretical DNA algorithm to solve the quadratic assignment problem using the Adleman-Lipton-sticker model, which was executed with an *O*(*kn*^4^) complexity and could handle the medium-sized cases. Nehi and Hamidi [13] corrected and further improved the DNA model proposed by Yang et al. [12]. Wang et al. [14] solved a traveling salesman problem by a DNA algorithm using the Adleman-Lipton model with an *O*(*n*) complexity. Based on the Adleman- Lipton model, Wang et al. [15] proposed a new DNA computing algorithm to tackle the capacitated vehicle routing problem with an *O*(*n*^2^) complexity.

In accordance with the processing order, the shop scheduling problem can typically be divided into three categories: the flow shop scheduling problem (FSSP), the job shop scheduling problem (JSSP) and the flexible job shop scheduling problem (FJSP). These three categories of problems are all about scheduling *n* jobs with varying processing times on *m* machines with varying speeds and capacities. In a JSSP, each job to be processed contains multiple operations, each of which is processed on a specified machine, and each job has a different machining path. In a FSSP, all jobs have the same machining path, i.e., the same operation sequence, without distinction between the operation and the machine. In a FJSP, the machining paths of the jobs are not necessarily the same and a job is allowed to be processed by any machine in a given set of machines. Many heuristic approaches have been developed to solve shop scheduling problems, such as particle swarm optimization (PSO), genetic algorithms (GA), simulated annealing (SA), tabu search (TS), artificial immune (AI), differential evolution algorithm (DEA), and ant colony optimization (ACO), among others, as well as their hybrids [16]. Mohamed Kurdi [17] proposed an effective genetic algorithm with a critical-path-guided Giffler and Thompson crossover operator (GA-CPG-GT) for JSSP. Zhou et al. [18] presented a hybrid social-spider optimization algorithm with a differential mutation (SSO-DM) operator to solve JSSP. Cruz-Chávez et al. [19] proposed a parallel algorithm that generated a set of parallel working threads, where each thread performed a simulated annealing process to solve JSSP. For JSSP, Pongchairerks [20] proposed a new two-level meta-heuristic algorithm composed of an upper-level algorithm and a lower-level algorithm.

However, due to the premature and local convergence of GA, its performance in dealing with complex JSSP is limited [21]. The particle swarm optimization (PSO) algorithm cannot effectively search the entire solution space, and may converge to a local optimal solution prematurely, and thus cannot achieve a good exploration- exploitation balance [22]. The quality of the optimal solution obtained by TS lies on the initial solution [23]. Due to the lack of memory function in SA, it may lead to repeated searches and easily fall into local optima, greatly affecting the effectiveness of SA and causing excessive search time [23]. Due to the dependence on random natural selection and recombination, the optimization results obtained by classical evolutionary algorithm are still limited [24]. Also due to the stubborn nature of JSSP, a single meta-heuristic method can no longer solve this problem well [18]. In addition, these heuristic approaches do not traverse all possible solutions, and can only find relatively good solutions through operations such as crossover, mutation and iteration. Even if a heuristic finds the optimal solution, the heuristic itself cannot prove that the solution it found is the actual optimal solution.

By contrast, DNA computing may be used to solve the JSSP. As long as appropriate encoding and manipulation are used, all possible solutions to the problem can be produced in one step. Deaton et al. [25] summarized three basic steps in using DNA computing to solve a problem: encoding, interaction and extraction. The first step is the basis of the other two steps, so that the key and the difficult part of DNA computing is to transform the problem into an equivalent DNA computing model by mapping.

Until now, there is not much reported research on solving JSSPs using DNA computing. Yin et al. [26] solved a FSSP using DNA computing by transforming the FSSP problem into a directed graph. Wang et al. [27] proposed a new parallel DNA algorithm to solve the task scheduling problem based on the Adleman-Lipton model, with an enlightening idea.

In this work, an appropriate encoding strategy is developed first to generate all possible solutions in parallel using DNA computing. The advantage of this encoding is that, once a scheduling sequence is determined, the makespan corresponding to each schedule is also determined. Theoretically efficient and parallel DNA algorithms based on Adleman-Lipton model are then proposed for solving the JSSP which can be solved with an *O*(*n*^2^) complexity. In the experiments, the DNA computing algorithms proposed in this work is simulated through two tool libraries of Python. Simulation experiments with 58 benchmark instances show that the proposed DNA algorithm outperforms other comparative heuristics.

The remainder of this paper is organized as follows. Section 2 describes the Adleman-Lipton model and describes the JSSP. Section 3 proposes a DNA algorithm for the JSSP and provides a performance analysis of the proposed DNA algorithm. Section 4 reports the experimental results of the proposed DNA algorithm and the comparison with several heuristic algorithms on 58 benchmark instances. Section 5 concludes this work with a summary and future research directions.

## 2. Preliminaries

This section is composed of two parts. The first part explains the Adleman-Lipton model, and the second part gives a formal description of the JSSP.

### 2.1 The Adleman-Lipton model

DNA is a polymer which is strung together from monomers called deoxyribonucleotides [28]. A single strand DNA molecule consists of a sequence of nucleotides with four different bases, i.e., adenine, guanine, cytosine and thymine, abbreviated as A, G, C and T, respectively. Every strand, according to its chemical structure, has a 5'-3' direction or a 3'-5' direction, with the 5'-end matching the 3'-end. In the double strand molecule, the two single strands have opposite directions. Using the Watson-Crick complementarity, i.e., the A-T pairing and the G-C pairing, without other possible pairings, a double strand DNA molecule can be formed under appropriate conditions. For instance, the single strand 5'-ACGTTA-3' and its complement 3'-TGCAAT-5' can form a double strand, also referred to as a duplex. Assume the upper strand runs from left to right in the 5'-3' direction, and consequently the lower strand runs from left to right in the 3'-5' direction. The complement 3'-TGCAAT-5' of the strand 5'-ACGTTA-3' is denoted by $\overset{¯}{\text{ACGTTA}}$. The length of a single strand DNA molecule is the number of nucleotides in the molecule. Thus a single strand consisting of 12 nucleotides is said to be a 12 mer, i.e., a polymer consisting of 12 monomers.

#### The Adleman-Lipton model

A test tube is a set of molecules of DNA, i.e., a multi-set of finite strings over the alphabets {A, G, C, T}. The following operations can be performed:

1. *Merge* (*N*_1_,*N*_2_,…,*N*_*k*_): given *k* test tubes *N*_1_,*N*_2_,…,*N*_*k*_, this operation pours the DNA solution in each of the test tubes *N*_2_,…,*N*_*k*_ into test tube *N*_1_. The uniform mixed solution is referred to as *N*_1_.
2. *Amplify*(*N*_1_,*N*_2_,…,*N*_*k*_): given a test tube *N*_1_, this operation creates copies of *N*_1_ and amplifies them into test tubes *N*_2_,…,*N*_*k*_.
3. *Separation* (*N*_1,_ *X*_,_ *N*_2_): given a test tube *N*_1_ and a string *X*, this operation transfers all the single strands containing string *X* in test tube *N*_1_ to test tube *N*_2_. The single DNA strands removed from test tube *N*_1_ are no longer contained in test tube *N*_1_. If *N*_1_ does not contain *X*, this operation does nothing.
4. *Selection* (*N*_1,_ *L*_,_ *N*_2_): given a test tube *N*_1_ and an integer *L*, this operation filters all DNA strands of length *L* in *N*_1_ and put them into test tube *N*_2_. Consequently, *N*_1_ no longer contains these filtered DNA strands.
5. *Append-head*(*N*,*S*): given a test tube *N* and a single strand *S*, this operation attaches (pastes) *S* to the front of every strand in *N*.
6. *Append-tail* (*N*,*R*): given a test tube *N* and a single strand *R*, this operation attaches (pastes) *R* to the end of every strand in *N*.
7. *Annealing* (*N*): given a test tube *N* with some single strands, this operation derives all possible double strands according to the *Watson-Crick complementarity* pairing principle, leaves them in *N*, and removes the other single strands from *N*.
8. *Denaturation* (*N*): given a test tube *N*, this operation separates every double-stranded DNA molecule into two single strands by heating without breaking the phosphodiester bond of each single strand. Briefly, the double-stranded DNA in *N* is separated as follows
  $$\left[\begin{array}{c} EF\\ \bar{EF} \end{array}\right]\Rightarrow [EF],[\bar{EF}].$$
9. *Cutting* (*N*, *ω*_1_*ω*_2_): given a test tube *N* and strings with *ω*_1_*ω*_2_, this operation divides every strand containing [*ω*_1_*ω*_2_] in *N* to different strands as follows
  $$[\cdots \alpha \omega_{1}\omega_{2}\beta \omega_{1}\omega_{2}\gamma \cdots ]\Rightarrow [\cdots \alpha \omega_{1}],[\omega_{2}\beta \omega_{1}],[\omega_{2}\gamma \cdots ],$$
  where *ω*_1_*ω*_2_ corresponds to the recognition site of the cutting operation.
10. *Discard* (*N*): given a test tube *N*, this operation clears all strands in *N*, that is, emptying *N*.
11. *Read* (*N*): given a test tube *N*, this operation obtains the precise DNA sequences of all strands in *N*.
12. *Sort* (*N*_1,_*N*_2,_*N*_3_): given a test tube *N*_1_ and two empty test tubes *N*_2_ and *N*_3_, this operation chooses the shortest strands in *N*_1_ and puts them in *N*_2_, chooses the longest strands in *N*_1_ and puts them in *N*_3_, and keeps the rest of the strands in *N*_1_.
13. *Ligation* (*N*): given a test tube *N*, this operation links all the DNA molecules (double strands) in *N* together by enzymes called ligases.
14. *Detect* (*N*): given a test tube *N*, this operation returns “*true*” if *N* contains at least one DNA strand, and returns “*false*” otherwise.
15. *T*: = *B*(*N*,*ω*): given a test tube *N* and a string *ω*, this operation produces the test tube *T* consisting of all strands in *N* which begin with the string *ω*.
16. *L*: = *Length*(*N*,*ω*,Ω): given a test tube *N*, this operation returns the length *L* of the specific single strand beginning with the string *ω* and ending with the string Ω in *N*.

In actual biological experiments, the above operations are feasible and achievable. Take the *Sort*(*N*_1_, *N*_2_, *N*_3_) operation as an example. In gel electrophoresis, the migration speed of DNA strands is related to its own length. The longer the strand, the slower the migration speed. Therefore, through gel electrophoresis experiments, the longest and shortest DNA strands in the test tube can be obtained. Since all operations mentioned above can be performed in lab within constant biological steps, it is reasonable to assume that the complexity of each operation is *O*(1). In previous works ([10–12, 14, 15, 27]), many researchers have used this same approach to analyze the complexity of DNA computing algorithms. Therefore, the same approach is used in this study.

### 2.2 The job shop scheduling problem

The JSSP is already known as a typical NP-hard problem [29]. An *n* × *m* JSSP can be formally described as follows [30]. There are *n* jobs and *m* machines denoted as *J* = (*J*_1_, *J*_2_, ⋯,*Jn*) and *M* = (*M*_1_, *M*_2_, ⋯,*M*_*m*_), respectively. Each job must be processed (or handled) through all *m* machines to fulfil its processing tasks. The processing of a job is also called an operation. Each job requires *m* operations. Only one machine is required for each operation, and only one operation can be handled on one of the *m* machines. Once started on a specified machine, an operation is not allowed to be interrupted until the processing of the job is completed, meaning that each operation begins only when all its previous operations are finished, i.e., preemption is not allowed. The processing time and the sequence of operations, i.e., the machining paths are given in advance. The goal of a JSSP is to find the optimal schedule in order to minimize the maximum makespan. A JSSP with *n* jobs and *m* machines has (*n*!)^*m*^ possible solutions.

The notations used for the mathematical description of the JSSP are given below.

1. *n* and *m* denote the numbers of jobs and machines, respectively.
2. *O*_*i*,*j*_ represents the operation *i* of job *j*, where *i* ∈ [1, *m*] and *j* ∈ [1, *n*].
3. *t*_*i*,*j*_ represents the processing time of *O*_*i*,*j*_.
4. *TJ*_*i*,*j*_ represents the completion time of *O*_*i*,*j*_, i.e., the cumulative completion time of job *j* up to operation *i*.
5. *TM*_*i*,*j*_ represents the earliest start time of *O*_*i*,*j*_, i.e., the cumulative time (not including *t*_*i*,*j*_) of machine *i* before job *j* starts.

The mathematical programming model of the JSSP is given in the following.

$$Min(\underset{1\leq j\leq n}{Max}(TJ_{m,j}))$$ (1)

Subject to

$$TJ_{i-1,j}+t_{i,j}\leq TJ_{i,j},\;\text{for}\;i\in \left[1,m\right]\;\text{and}\;j\in \left[1,n\right]$$ (2)

$$TM_{i,j}+t_{i,j}\leq TJ_{i.j},\;\text{for}\;i\in \left[1,m\right]\;\text{and}\;j\in \left[1,n\right]$$ (3)

$$TJ_{ij}\geq 0,\;TM_{ij}\geq 0,\;\text{for}\;i\in \left[1,m\right]\;\text{and}\;j\in \left[1,n\right].$$ (4)

The objective function minimizes the makespan, i.e., the maximum completion time. Constraint (2) represents precedence relationship between the operations. Constraint (3) means that preemption is not allowed. Constraint (4) gives the domains of the variables.

#### Example 1

Table 1 shows a *n* × *m* = 4 × 2 FSSP example with 4 jobs *J*_1_, *J*_2_, *J*_3_ and *J*_4_ and 2 machines *M*_*1*_ *M*_1_ and *M*_2_. The jobs have the same operation sequence, i.e., the same machining path, where they first pass through machine 1 (*M*_1_) and then pass through machine 2 (*M*_2_). The time needed by each job on each machine is shown in the table.

**Table 1:** A *n* × *m* = 4 × 2 FSSP.

Machine	Job			
	*J*_1_	*J*_2_	*J*_3_	*J*_4_
*M*_1_	15	8	6	12
*M*_2_	4	10	5	7

#### Example 2

Table 2 shows a *n* × *m* = 3 × 3 JSSP example with 3 jobs *J*_1_, *J*_2_ and *J*_3_, each with a different machining path, processed on 3 machines, *M*_1_, *M*_2_ and *M*_3_. The machines required are shown in the column *M*_*i*_ and the time needed by each job on each machine is shown in the column *t*_*i*,*j*_. For instance, the 1^st^ operation of job 1 (*J*_1_), i.e., *O*_1,1_, is processed on machine 3 (*M*_3_) and the processing time corresponding to *O*_1,1_ is *t*_1,1_ = 7 units. The 2^nd^ operation of job 1 (*J*_1_), i.e., *O*_2,1_, is processed on machine 1 (*M*_1_) and the processing time corresponding to this operation is *t*_2,1_ = 4 units, and so on.

**Table 2:** A *n* × *m* = 3 × 3 JSSP.

*i*	*J*_1_		*J*_2_		*J*_3_	
	*M*_*i*_	*t*_*i*,1_	*M*_*i*_	*t*_*i*,2_	*M*_*i*_	*t*_*i*,3_
1	3	7	2	5	2	4
2	1	4	3	6	1	2
3	2	2	1	3	3	3

From the two examples above, it is intuitive that the JSSP is an extension of the FSSP. The biggest difference between a JSSP and a FSSP lies in the machining paths of the jobs, as shown in Tables 1 and 2. As compared with the FSSP as shown in Table 1, the machining paths of the jobs are different from each other in a JSSP as shown in Table 2. If all the jobs have the same machining path, i.e., each job needs the same operations, without distinction between the operations and the machines, the JSSP becomes a FSSP.

As compared with FSSP, JSSP is much more complicated and closer to the practical problems in production. In a FSSP, only one time matrix is needed. However, in a JSSP, two matrices are required, one represents the processing time and the other represents the machines needed by the jobs. Therefore, this work focuses on the more practical and representative JSSP for an in-depth study.

## 3. A DNA algorithm for the job shop scheduling problem

Encoding is the key and difficult part of solving the combinatorial optimization problem with DNA computing. Therefore, this section starts with the coding scheme and then gives an overview of the proposed algorithm. The detailed algorithm is finally presented.

### 3.1 Encoding

A schedule, or scheduling sequence, of an *n* × *m* JSSP can be denoted by *OP*_1_−*OP*_2_−⋯*OP*_*n×m*_, where *OP*_*i*_∈[1, *n*] indicates a job number. In this schedule, the *i*^*th*^ appearance of job *j* indicates operation *i* of job *j*, i.e., *O*_*i*,*j*_. Each number *OP*_*i*_ appears exactly *m* times.

**Fig 1:**
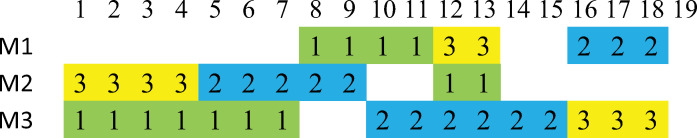
A Gantt chart for the schedule 1-3-2-2-1-3-3-1-2 of Example 2.

Take a scheduling sequence 1-3-2-2-1-3-3-1-2 of a 3 × 3 JSSP as an example. The first number ‘1’ indicates operation 1 of job 1; the second number ‘3’ indicated operation 1 of job 3. The fourth number ‘2’ (the 2^nd^ appearance of job 2) indicates operation 2 of job 2. Similarly, the seventh number ‘3’ (the 3^rd^ appearance of job 3) indicates operation 3 of job 3. Obviously, once a scheduling sequence is determined, the makespan corresponding to this schedule is uniquely determined. For example, referring to the data in Table 2 in Example 2, the completion times or makespans of the 3 jobs can be easily calculated. The completion times of jobs 1, 2 and 3 are 13, 18 and 18, respectively. Consequently, the makespan (completion time) for this schedule is 18. Fig 1 in the following shows this schedule as a Gantt chart.

Effective encoding needs to be used to reasonably map real problems to DNA molecular computing models, and to generate all possible solutions in parallel in one step. In the following, *p*, *E*_*i*_, *q*, *F*_*j*_ are used to represent different DNA single strands with the same length, e.g., *u* mer, with *u* as a positive integer. The notations *p* and *q* are used for DNA ligation, as defined in Section 2.2, and *E*_*i*_ and *F*_*j*_ represent the single strands for operation *i* and job *j*, respectively. The single strand *pE*_*i*_*qF*_*j*_ can be used to indicate operation *i* of job *j*, i.e., *O*_*i*,*j*_. In this way, all ${\left(n!\right)}^{m}$ possible solutions can be easily generated.

#### Example 3

Table 3 shows a *n* × *m = 5* × 6 JSSP example with 5 jobs, each with a different machining path, processed on 6 machines. Data for each job are shown in two columns in the table. The first column shows the corresponding machine number and the second column shows the time needed by the job on each machine.

**Table 3:** A *n* × *m = 5* × 6 JSSP.

*i*	*J*_1_		*J*_2_		*J*_3_		*J*_4_		*J*_5_	
	*M*_*i*_	*t*_*i*,1_	*M*_*i*_	*t*_*i*,2_	*M*_*i*_	*t*_*i*,3_	*M*_*i*_	*t*_*i*,4_	*M*_*i*_	*t*_*i*,5_
1	3	3	2	6	3	1	4	7	5	6
2	1	10	3	8	4	5	1	4	2	10
3	2	9	5	1	6	5	3	4	3	7
4	4	5	6	5	1	5	2	3	6	8
5	6	3	1	3	2	9	5	1	1	5
6	5	10	4	3	5	1	6	3	4	4

**Fig 2:**
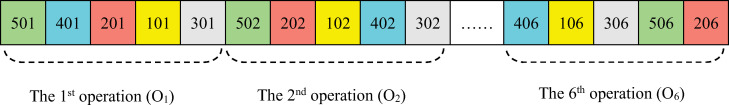
An optimal schedule of the 5×6 JSSP in Example 3.

Fig 2 in the following is an optimal scheduling sequence of this 5×6 JSSP. It means that the jobs are processed in the order of 5-4-2-1-3 for the 1^st^ operation, in the order of 5-2-1-4-3 for the 2^nd^ operation, and so on, where the number ‘0’ in the middle represents a separator. The same job processed by different machines are identified with the same color. A DNA encoding method based on scheduling sequences is proposed below.

In accordance with *Algorithm* 1, the DNA strands {*pE*_1_*qF*_5_*pE*_1_*qF*_4_*pE*_1_*q- F*_2_*pE*_1_*qF*_1_*pE*_1_*qF*_3_} will be generated to denote that the jobs are processed in the order of 5-4-2-1-3 in the 1^st^ operation. In this way, DNA strands can be obtained to denote all *n* jobs in every operation. By encoding the jobs in this manner, all (*n*!)^*m*^ possible schedules will be obtained.

**Fig 3:**
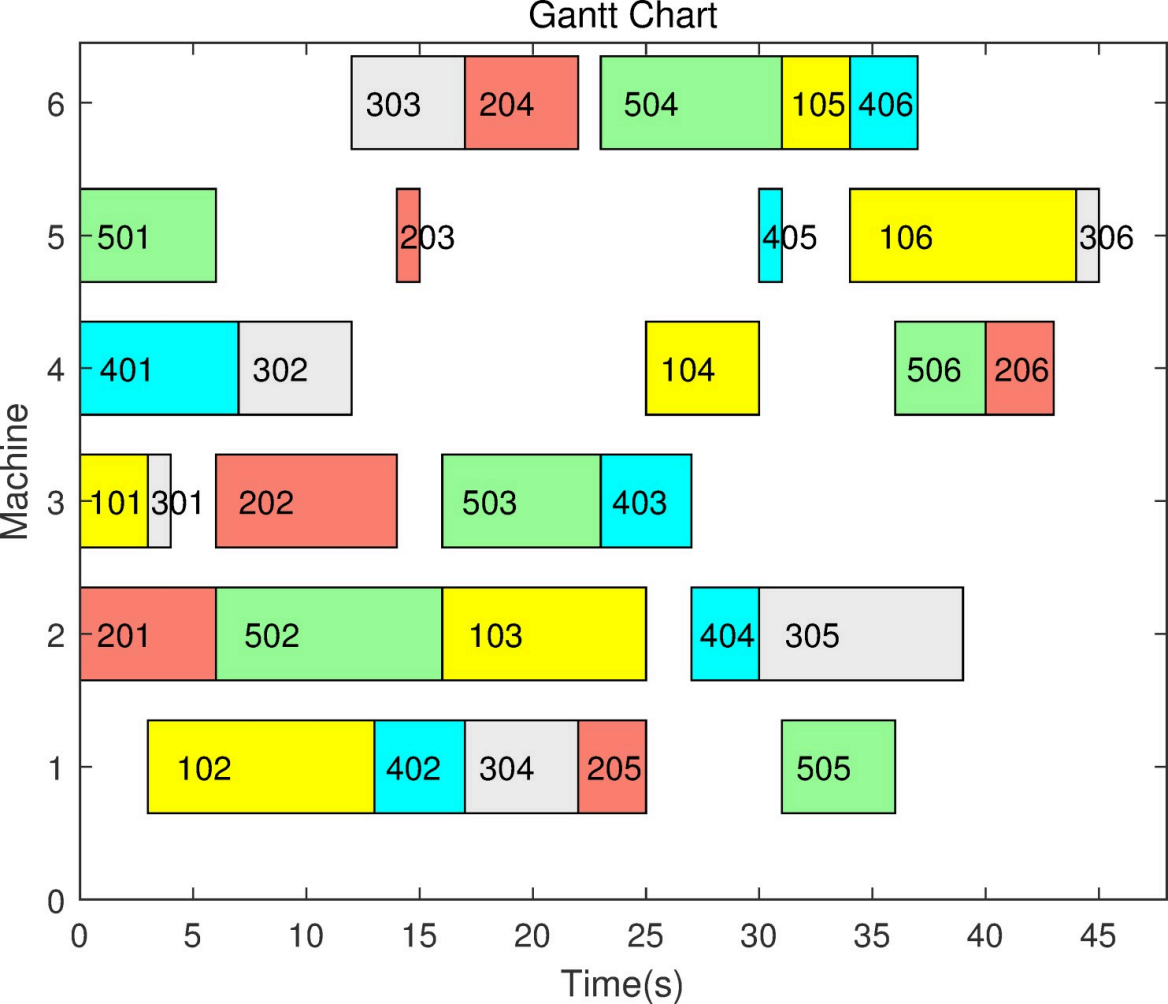
Gantt chart of an optimal schedule of the 5×6 JSSP in Example 3.

Fig 3 in the following is the optimal schedule presented as a Gantt chart corresponding to the optimal scheduling sequence given above in Fig 2, where the maximum completion time, i.e., the makespan, of this schedule is 45. The result 45 is calculated using the data in Table 3.

In JSSP, different jobs may require the same machine in some operations, so that some jobs may have to wait for others to finish before being processed and machines may become idle while having to wait for jobs to come. Different schedules have different job and machine waiting times and might have different makespans. The advantage of this encoding is that, once a scheduling sequence, i.e., a schedule, is determined, the makespan corresponding to this schedule is also uniquely determined. However, it should be noted that the makespan is, but the corresponding scheduling sequences may not be, unique.

### 3.2 An outline of the algorithm

The basic idea of this DNA algorithm for solving the JSSP is to find an optimal solution by checking all possible solution candidates. This brute force approach is realized through DNA computing. Specifically, this proposed algorithm consists of four steps.

1. Generate the initial solution space in test tube *N*_0_ for the JSSP;
2. Screen the DNA strands representing the feasible schedules and discard the ones representing infeasible schedules;
3. Append time information strands at the end of the strands representing feasible schedules and calculate the completion time of each feasible schedule;
4. Select the strands corresponding to the optimal schedule that minimizes the maximum completion time, i.e., the makespan.

**Fig 4:**
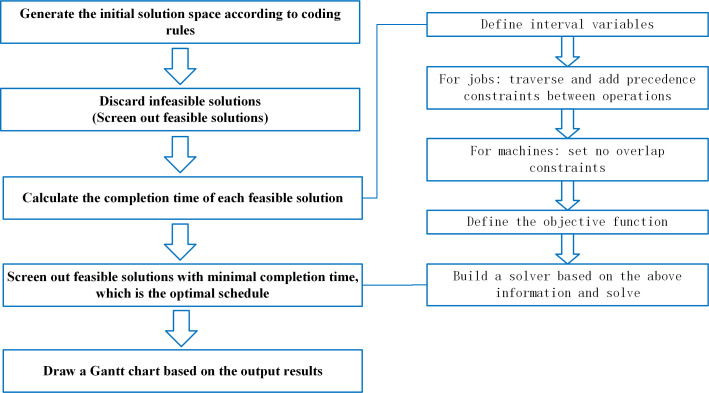
Algorithm flow chart.

The flowchart of the algorithm is shown in Fig 4 as follows.

### 3.3 Detailed algorithm

#### 3.3.1 Initialization of the sequence of the *n* jobs for each operation

Initial test tube:

$$N_{i}=\left\{pE_{i}q\right\}\;\text{for}\;1\leq i\leq m$$

$$Q=\{F_{1},F_{2},\cdots ,F_{n},\bar{qF_{1}},\bar{qF_{2}},\cdots ,\bar{qF_{n}},\bar{qF_{1}p},\bar{qF_{2}p},\cdots ,\bar{qF_{n}p}\}$$

### Algorithm 1. Initialization of the sequence of the *n* jobs for each operation

Begin

1: **for** *i =* 1 *to m* **do**

2:   *Merge*(*N*_*i*_, *Q*);

3:  *Annealing*(*N*_*i*_);

4:  *Ligation*(*N*_*i*_);

5:  *Denaturation*(*N*_*i*_);

6:  *Selection*(*N*_*i*_, 4*nu*, *V*_*i*_);

7:  *Discard*(*N*_*i*_);

8:  *V*_*i*_: = *B*(*T*_*i*_, *pE*_*i*_*q*);

9:  **for** *j =* 1 *to j = n* **do**

10:   *Separation*(*V*_*i*_, *qF*_*j*_*p*, *U*_*i*_);

11:   *Discard*(*V*_*i*_);

12:   *Amplify*(*U*_*i*_, *V*_*i*_);

13:   *Discard*(*U*_*i*_);

14:  **end for**

15:  *Amplify*(*V*_*i*_, *N*_*i*_);

16:  *Discard*(*V*_*i*_);

17:  *Append-tail*(*N*_*i*_, *a*_i2_*S*);

18:  *Append-head*(*N*_*i*_, *a*_i1_);

19: **end for**

End

Algorithm 1 produces DNA strands representing all *n* jobs for every possible operation, for instance,

$$\begin{array}{c} \left\{a_{11}pE_{1}qF_{2}pE_{1}qF_{4}pE_{1}qF_{5}pE_{1}qF_{1}pE_{1}qF_{3}a_{12}S\right\}\\ \text{and}\left\{a_{21}pE_{2}qF_{2}pE_{2}qF_{1}pE_{2}qF_{5}pE_{2}qF_{4}pE_{2}qF_{3}a_{22}S\right\}, \end{array}$$

and so on. The lengths of the single strands *a*_*i*,*j*_ and *S* are also *u* mer. The strands *a*_*i*,*j*_ and *S* are used for connection in the following algorithm.

#### 3.3.2 Generation of all possible strands for the JSSP

Initial test tube *N*_0_:

$$N_{0}=\{\bar{a_{12}Sa_{21}},\bar{a_{22}Sa_{31}},\bar{a_{32}Sa_{41}},\cdots ,\bar{a_{m-1,2}Sa_{m1}}\}$$

### **Algorithm 2.** Generation of all possible strands for the JSSP

Begin

1: *Merge* (*N*_0_, *N*_1_, *N*_2_,…, *N*_*m*_);

2: *Annealing* (*N*_0_);

3: *Denaturation* (*N*_0_);

4: *N*_0_: = *B* (*N*_0_, *a*_11_*pE*_1_*q*);

5: *Selection* (*N*, (4*n*+3)*mu*, *N*_0_);

6: **for** *i =* 1 *to i = m* **do**

7:  *Separation* (*N*_0_, *a*_i2_*S*, *N*_1_);

8:  *Discard* (*N*_0_);

9:  *Amplify* (*N*_1_, *N*_0_);

10:  *Discard* (*N*_1_);

11: **end for**

End

After executing Algorithm 2, all possible DNA strands representing all possible solutions of the JSSP can be obtained as shown below

$$\{a_{11}pE_{1}qF_{j_{1}}\cdots pE_{1}qF_{j_{k}}\cdots pE_{1}qF_{j_{n}}a_{12}Sa_{21}pE_{2}qF_{j_{1}}\cdots pE_{2}qF_{j_{k}}\cdots pE_{2}qF_{j_{n}}a_{22}S\cdots \},$$

where the subscript *j*_*k*_ of $F_{j_{k}}$ is uniquely determined by the value of *k* for 1 ≤ *k* ≤ *n*, and $F_{j_{k}}\in \{F_{1},F_{2},\cdots ,F_{n}\}$, i.e., the sequence *j*_1_, …,*j*_*k*_, …,*j*_*n*_ is an arbitrary out-of-order combination of the sequence 1,…,*n*.

#### 3.3.3 Computation of the final completion time of each job for every strand

In Algorithm 3, as explained in Section 2.2, $TJ_{i,j_{k}}$ denotes the completion time of job *j*_*k*_ in operation *i*, $TM_{i,j_{k}}$ is the cumulative time (not including $t_{i,j_{k}}$) of the required machine corresponding to job *j*_*k*_ in operation *i*, and $t_{i,j_{k}}$ is the corresponding processing time of job *j*_*k*_ in operation *i*. The value of *j*_*k*_ is also uniquely determined by the value of *k* for 1 ≤ *k* ≤ *n*, where *j*_*k*_ ∈{1,2,⋯,n}. The final completion time of the *n* jobs are stored separately in *n* test tubes. The single strand *Ψ*, also with a length of *u* mer, in Algorithm 3 denotes one unit of time.

### Algorithm 3. Computation of the final completion time of each job for every strand

Begin

1: *Amplify* (*N*_0_,*N*_1_,*N*_2_, …,*N*_*n*_);

*2*: *Discard (N*_0_*);*

3: **for** *i* = 1 *to* ***m* do**

4:  **for** *k =* 1 *to* ***n* do**

5:   **if** *i*>1 **then**

6:    *Separation* ($N_{j_{k}}$,*Sω*, $U_{j_{k}}$);

7:    *Discard* ($N_{j_{k}}$);

8:    *Cutting* ($U_{j_{k}}$,*Sω*);

9:    $N_{j_{k}}$: *= B* ($U_{j_{k}}$,*a*_11_*pE*_1_*q*);

10:    *Discard* ($U_{j_{k}}$);

11:   **else**

12:    *Continue*

13:   **if** *$TJ_{i-1,j_{k}}>TM_{i,j_{k}}$*(*when i* = 1, *both initial values are* 0) **then**

14:    *Append-tail* ($N_{j_{k}}$, $\omega \overset{\Psi \Psi \cdots \Psi \Psi }{\underset{TJ_{i-1,j_{k}}+t_{i,j_{k}}}{⏟}}\Omega$);

15:   **else**

16:    *Append-tail* ($N_{j_{k}}$, $\omega \overset{\Psi \Psi \cdots \Psi \Psi }{\underset{TM_{i,j_{k}}+t_{i,j_{k}}}{⏟}}\Omega$);

17:   $TJ_{i,j_{k}}$: = *Length*($N_{j_{k}}$, *ω*, Ω);

18:   $TM_{i,j_{k}}$: = *Length*($N_{j_{k}}$, *ω*, Ω);

19:  **end for**

20: **end for**

End

#### 3.3.4 DNA optimization

This algorithm finds the optimal schedule that minimizes the maximum completion time, i.e., the makespan,

$$Min(\underset{1\leq j\leq n}{Max}(TJ_{m,j})),$$

where *TJ*_*m*,*j*_ is the final completion time of job *j* in the last operation.

### Algorithm 4. DNA optimization

Begin

1: **for** *i =* 1 *to n* **do**

2:  *Sort* (*N*_*i*_, *V*_1_, *V*_2_);

3: **end for**

4: *Sort* (*V*_2_, *V*_0_, *V*_3_);

5: *Cutting* (*V*_0_, *Sω*);

6: *T*_0_: = *B* (*V*_0_, *a*_11_*pE*_1_*q*);

7: *Read* (*V*_0_)

End

Theorem in the following is obtained by inspecting Algorithms 1–4 line by line.

#### Theorem

*Without loss of generality*, *n ≥ m is assumed*. *The solutions of a n×m JSSP has an O(n*^*2*^*) complexity using DNA computing*.

#### Proof

The total complexity of the four algorithms is as follows

$$O\left(Algorithm1\right)=O\left(m\left(7+4n+4\right)\right)\approx O\left(4n^{2}+11n\right)=O\left(n^{2}\right);$$

O(Algorithm2)=O(5+4m)≈O(4n+5)=O(n);

$$O\left(Algorithm3\right)=O\left(10mn+2\right)\approx O\left(10n^{2}+2\right)=O\left(10n^{2}\right)=O\left(n^{2}\right);$$

O(Algorithm4)=O(n+4)=O(n);

$$\begin{array}{l} O=O\left(Algorithm1\right)+O\left(Algorithm2\right)+O\left(Algorithm3\right)+O\left(Algorithm4\right)\\ \;=O\left(n^{2}\right)+O\left(n\right)+O\left(n^{2}\right)+O\left(n\right)=O\left(n^{2}\right) \end{array}$$

In conclusion, the optimal schedule of a JSSP can be found with an *O*(*n*^2^) complexity.

#### Summary

The solution of the JSSP can be represented by a strand with a polynomial length.

#### Explanation

Suppose the length of the different strands is

$$||E_{i}||=||F_{j}||=||a_{ij}||=||S||=||\Psi ||=||\Omega ||=||\omega ||=||p||=||q||=u\;\text{mer},\;\text{for}\;i\;\in [1,\;m]\;\text{and}\;j\;\in \;[1,\;n].$$

Let *l* = ΣΣ*t*_*ij*_, and also assume *m* ≤ *n*. The length of DNA strand *L* corresponding to the optimal schedule in Algorithm 4 is as follows.

$$\begin{array}{l} \left||L||=n\sum_{i=1}^{m}\left||E_{i}|\right|+m\sum_{j=1}^{n}\left||F_{j}|\right|+mn(||p||+||q||)+||\Omega ||+||\omega |\right|\\ \begin{array}{cc} & +\overset{\text{||}\Psi \text{||}+\cdots +\text{||}\Psi \text{||}}{\underset{Min(\underset{1\leq j\leq n}{Max}(TJ_{m,j}))<<l}{⏟}}+\sum_{i=1}^{m}\left(||a_{i1}||+||a_{i2}||\right)+m||S|| \end{array}\\ <mnu+mnu+mn2u+lu+2mu+mu\\ <n^{2}u+n^{2}u+2n^{2}u+lu+2nu+nu\\ =(4n^{2}+3n+l)u \end{array}$$

The final solution strand in Algorithm 4 is within appropriate length. The optimal solution can then be found and determined.

## 4. Experiment and comparison

The algorithm proposed in this study is simulated in Python. Two important tool libraries, i.e., Biopython and DOcplex, are used to simulate and implement the four algorithms, as components of the proposed algorithm, in this work. Biopython, a Python tool for computational molecular biology, is used to encode problems and construct solution spaces. DOcplex, a Python tool library for solving constraint programming problems, is used to simulate the constraints in Algorithm 3 and the objective function in Algorithm 4. The computer used for computation has an i5-4210H processor with a 2.90GHz clock speed and 12G of RAM.

The algorithm is first compared with four state-of-the-art heuristics on 43 JSSP benchmark instances (see Table 4). The results show that, except for instance LA29, the proposed algorithm found the best known solutions for the remaining 42 instances, and has the same or better performance than the four comparative heuristics.

**Table 4:** Results obtained by the proposed algorithm and four comparative heuristics for the 43 instances.

Instances	Size	BKS	Proposed	MAGATS	NIMGA	aLSGA	WW
FT06	6×6	55	**55**	55	55	55	55
FT10	10×10	930	**930**	930	930	930	930
FT20	20×5	1165	**1165**	1165	**1173**	1165	1165
LA01	10×5	666	**666**	666	666	666	666
LA02	10×5	655	**655**	655	655	655	655
LA03	10×5	597	**597**	597	597	**606**	597
LA04	10×5	590	**590**	590	590	**593**	590
LA05	10×5	593	**593**	593	593	593	593
LA06	15×5	926	**926**	926	926	926	926
LA07	15×5	890	**890**	890	890	890	890
LA08	15×5	863	**863**	863	863	863	863
LA09	15×5	951	**951**	951	951	951	951
LA10	15×5	958	**958**	958	958	958	958
LA11	20×5	1222	**1222**	1222	1222	1222	1222
LA12	20×5	1039	**1039**	1039	1039	1039	1039
LA13	20×5	1150	**1150**	1150	1150	1150	1150
LA14	20×5	1292	**1292**	1292	1292	1292	1292
LA15	20×5	1207	**1207**	1207	1207	1207	1207
LA16	10×10	945	**945**	945	945	**946**	945
LA17	10×10	784	**784**	784	784	784	784
LA18	10×10	848	**848**	848	848	848	848
LA19	10×10	842	**842**	842	842	**852**	842
LA20	10×10	902	**902**	**907**	**907**	**907**	**907**
LA21	15×10	1046	**1046**	1046	**1058**	**1068**	**1046**
LA22	15×10	927	**927**	927	**937**	**956**	**935**
LA23	15×10	1032	**1032**	1032	1032	1032	1032
LA24	15×10	935	**935**	935	**947**	**966**	**937**
LA25	15×10	977	**977**	977	**989**	**1002**	977
LA26	20×10	1218	**1218**	1218	1218	**1223**	1218
LA27	20×10	1235	**1235**	1235	**1269**	**1281**	**1236**
LA28	20×10	1216	**1216**	1216	**1247**	**1245**	1216
LA29	20×10	1152	**1176**	**1164**	**1241**	**1230**	**1160**
LA30	20×10	1355	**1355**	1355	1355	1355	1355
LA31	30×10	1784	**1784**	1784	1784	1784	1784
LA32	30×10	1850	**1850**	1850	1850	1850	1850
LA33	30×10	1719	**1719**	1719	1719	1719	1719
LA34	30×10	1721	**1721**	1721	1721	1721	1721
LA35	30×10	1888	**1888**	1888	1888	1888	1888
LA36	15×15	1268	**1268**	**1281**	**1293**	**–**	**1279**
LA37	15×15	1397	**1397**	1397	**1432**	**–**	**1407**
LA38	15×15	1196	**1196**	**1198**	**1222**	**–**	1196
LA39	15×15	1233	**1233**	1233	**1251**	**–**	**1242**
LA40	15×15	1222	**1222**	**1228**	**1246**	**–**	**1229**

The 43 JSSP benchmark instances are selected from the OR Library [31], which contains 3 instances (FT06, FT10, FT20) designed by Fisher and Thompson [32] and 40 instances (LA01~LA40) designed by Lawrence [33]. The four comparative heuristics used for comparison are MAGATS [21], NIMGA [34], aLSGA [35] and WW [36].

Table 4 shows the results obtained by the proposed algorithm and the four comparative heuristics for the 43 instances. These results include the names of the instances, the sizes of the instances represented by *n*×*m*, the best known solutions (BKS) and the best solutions obtained by the proposed algorithm and the four comparative heuristics. Results of the comparative heuristics are from the original respective publications [21, 34–36].

**Fig 5:**
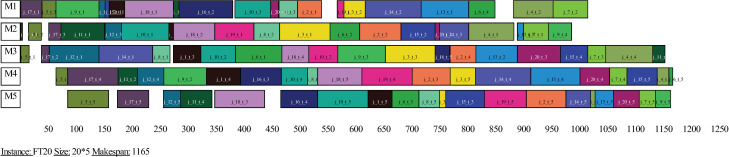
Gantt chart of an optimal schedule of instance FT20.

**Fig 6:**
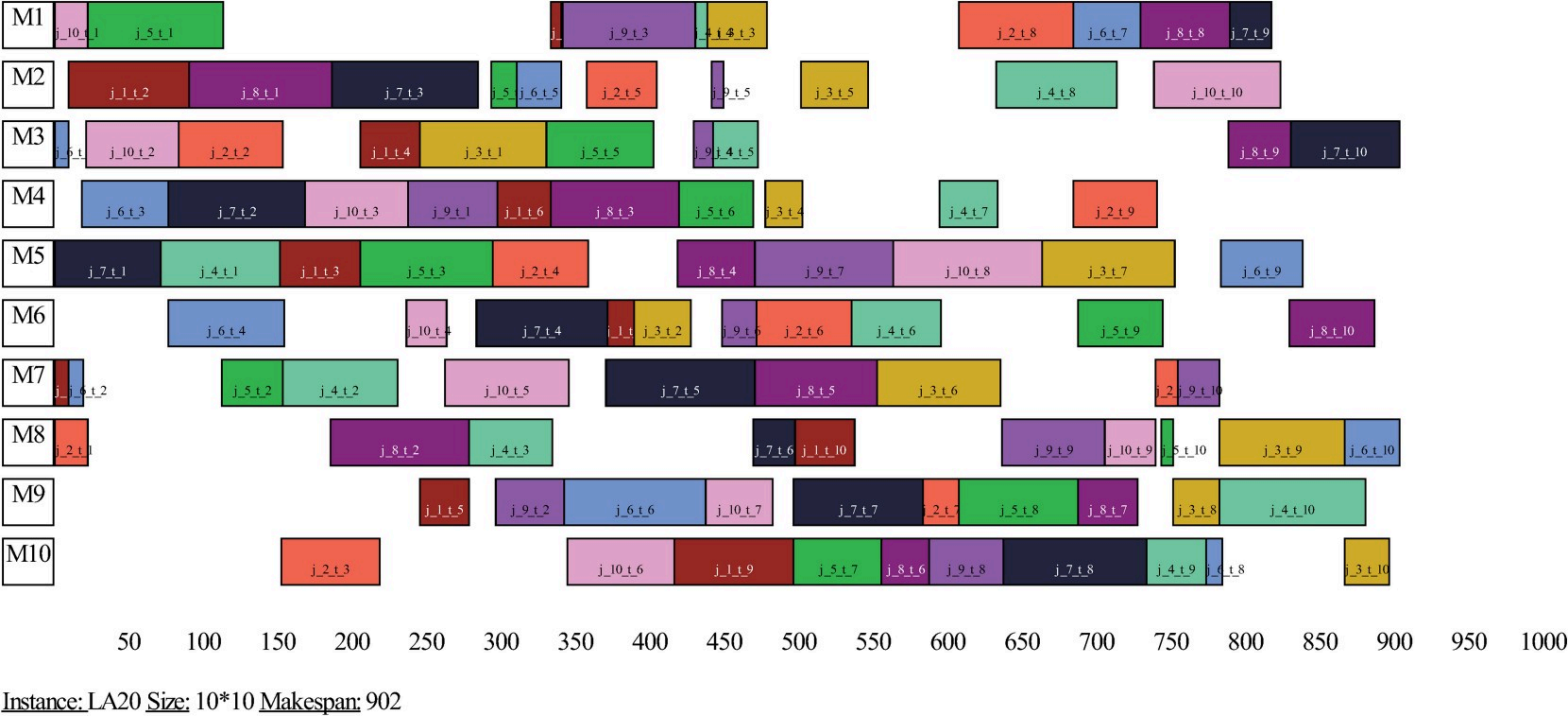
Gantt chart of an optimal schedule of instance LA20.

**Fig 7:**
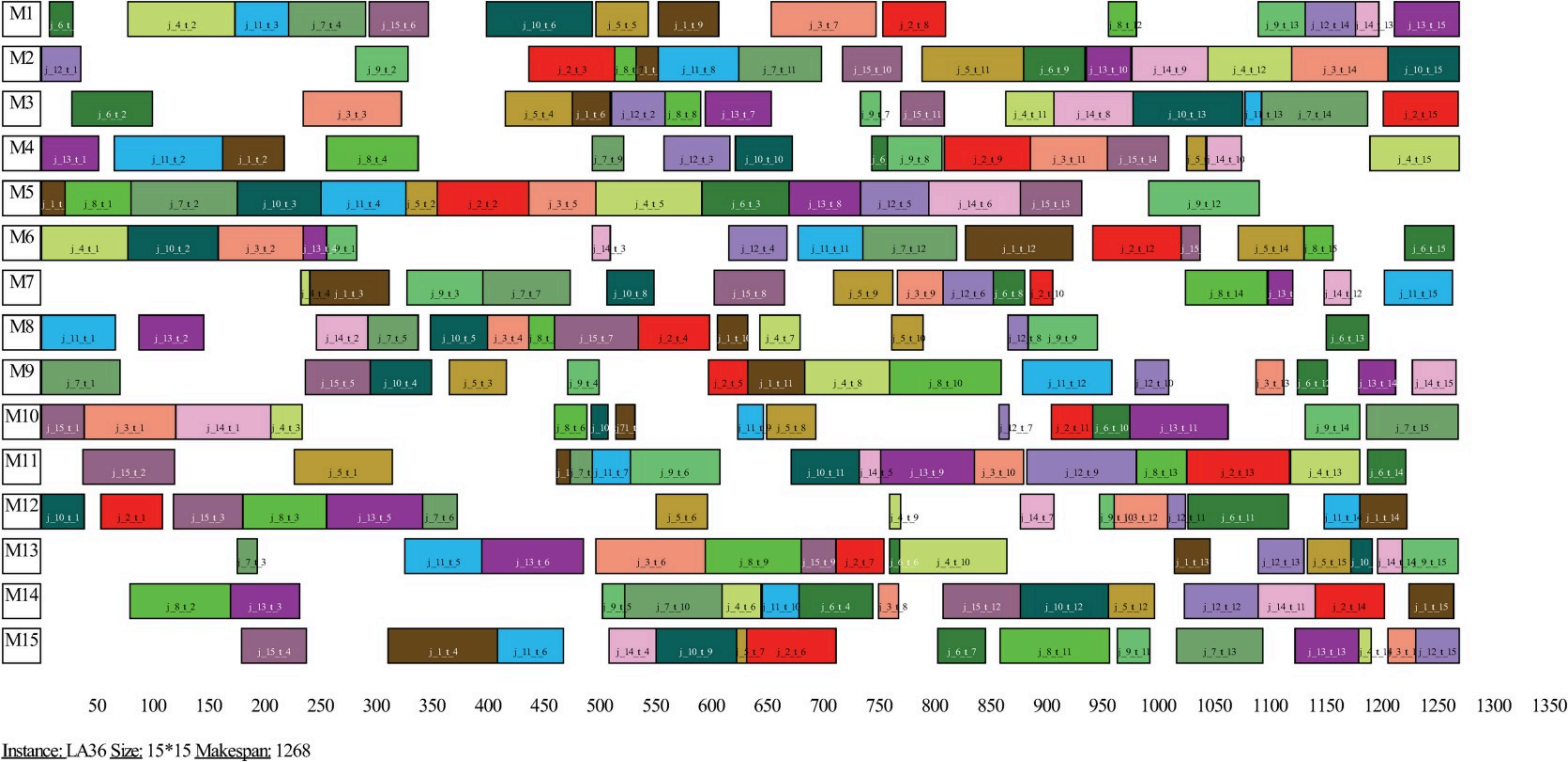
Gantt chart of an optimal schedule of instance LA36.

In order to visualize the scheduling results of the proposed algorithm, the Gantt charts of the optimal schedules of the instances FT20, LA20 and LA36 are presented in Figs 5–7, respectively. Figs 5–7 show that the optimal makepans of instances FT20, LA20 and LA36 are 1165, 902 and 1268 units of time, respectively. Not all four comparative heuristics could find the best known solution for instance FT20 and none of these heuristics could find the best known solutions for instances LA20 and LA36.

**Fig 8:**
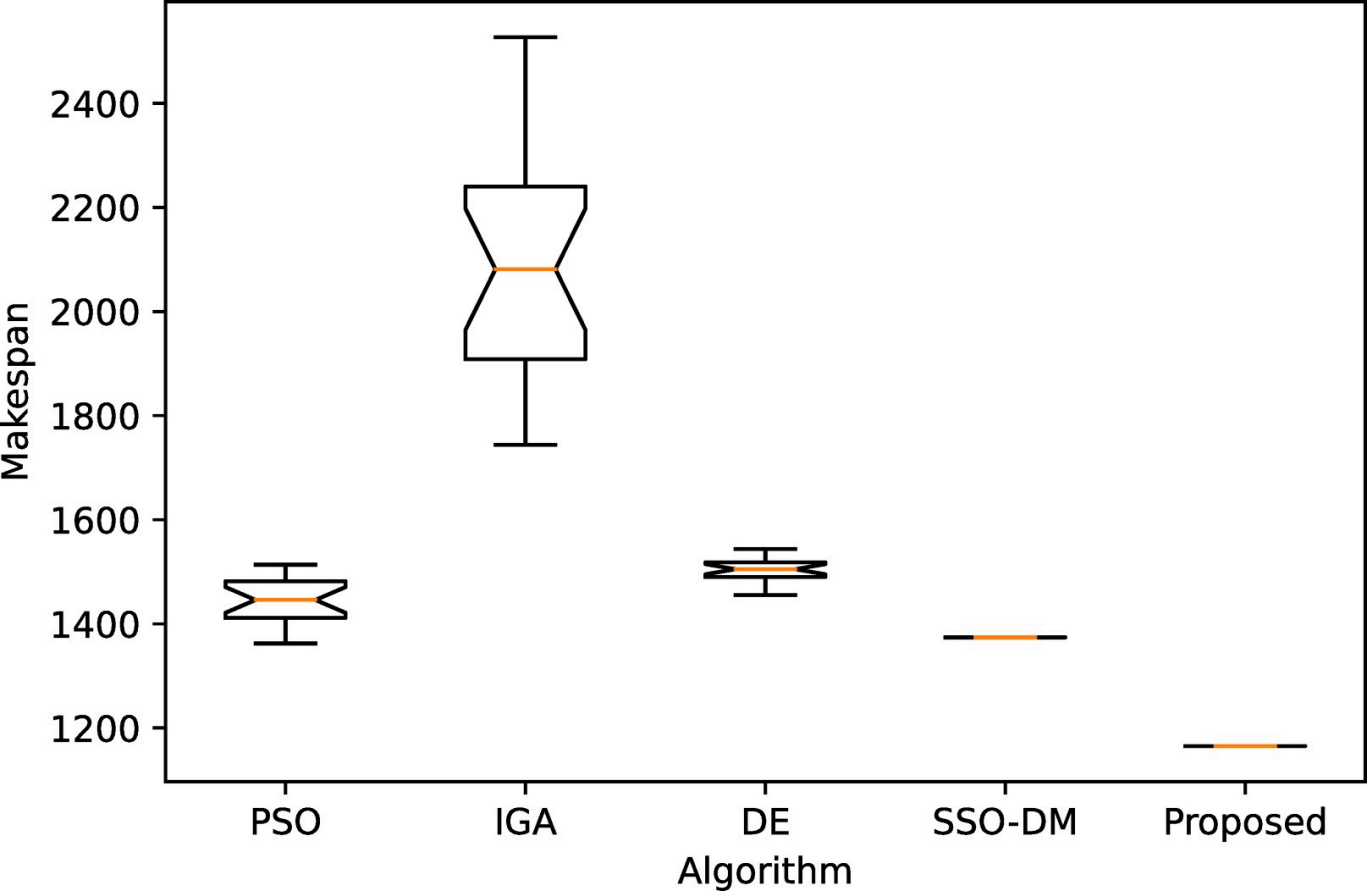
The box plot for FT20.

**Fig 9:**
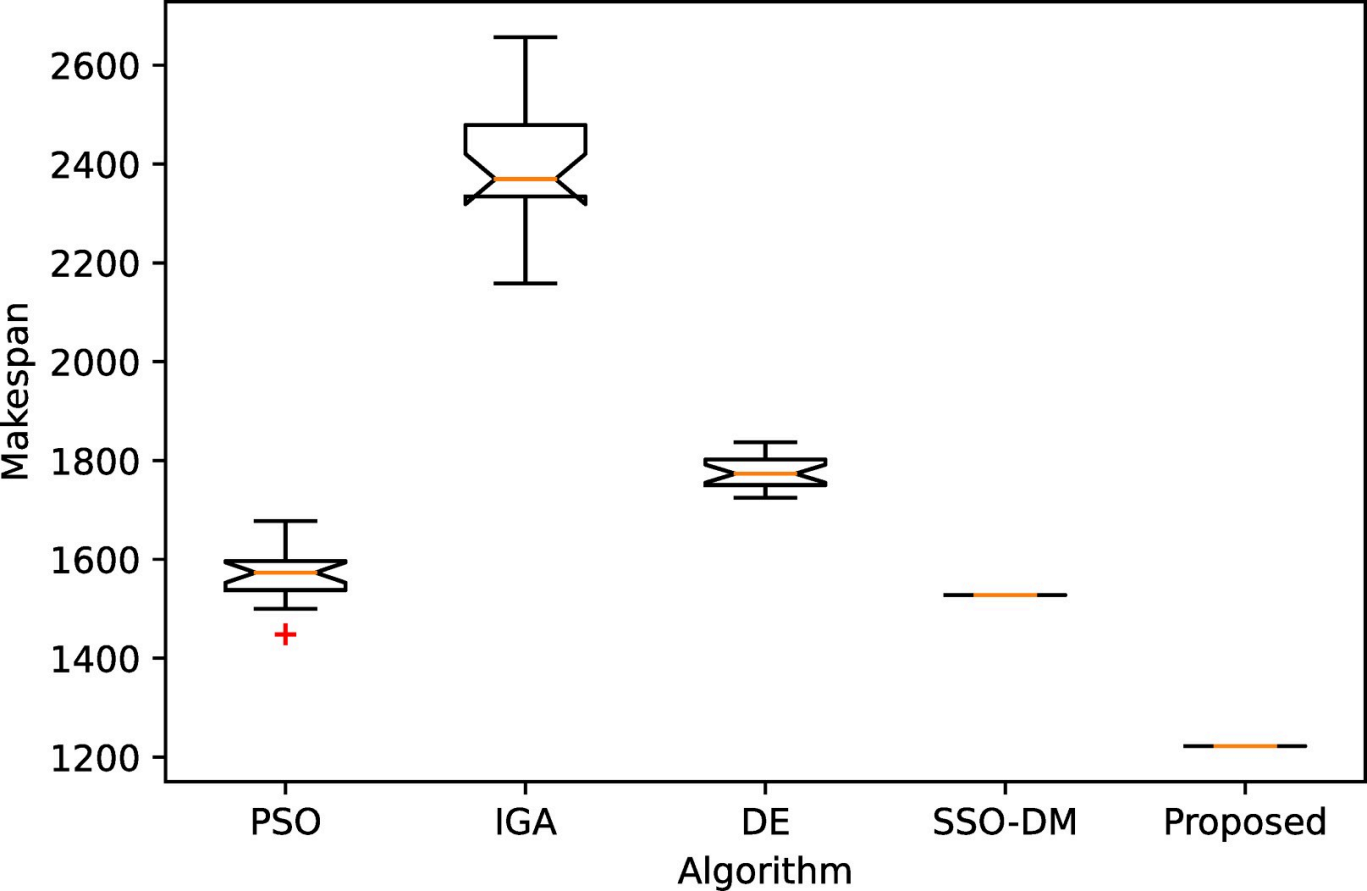
The box plot for LA40.

**Fig 10:**
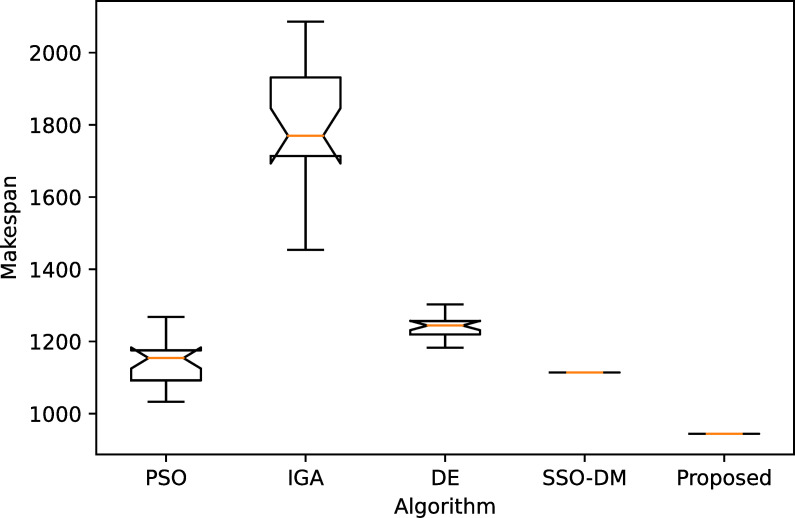
The box plot for ORB10.

**Fig 11:**
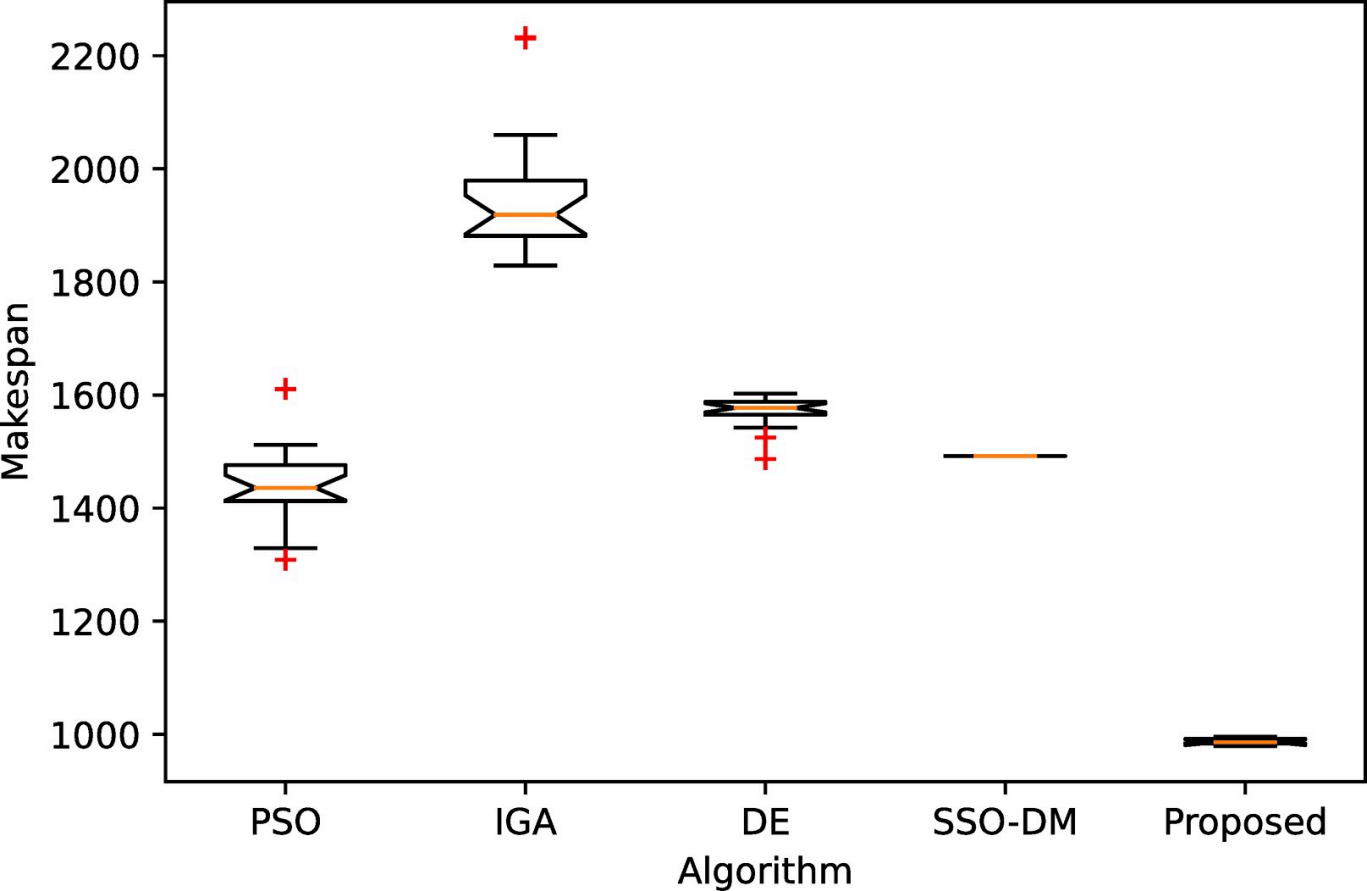
The box plot for YN4.

Table 5 shows a comparative analysis with four more heuristics on four instances of different sizes. The four heuristics are PSO [37], IGA [38], DE [39] and SSO-DM [18]. The table gives the statistical results, i.e., the best, worst, mean and standard deviation (Std.), of 20 independent runs. Except for instance YN4, the proposed algorithm found the best known solutions for the remaining three instances. The results of the comparative heuristics are from Zhou et al. [18]. Figs 8–11 show the box plots of these five algorithms on the four instances.

**Table 5:** Statistical results of five algorithms on four instances.

Instances	Size	BKS	Algorithm	Best	Worst	Mean	Std.
FT20	20×5	1165	PSO	1374.00	1521.00	1442.50	42.02
			IGA	1744.00	2527.00	2025.50	198.95
			DE	1456.00	1554.00	1506.00	27.64
			SSO-DM	1374.00	1374.00	1374.00	0
			Proposed	1165.00	1165.00	1165.00	0
LA40	15×15	1222	PSO	1498.00	1732.00	1576.05	59.79
			IGA	2154.00	2803.00	2340.25	155.90
			DE	1691.00	1824.00	1767.05	36.46
			SSO-DM	1528.00	1528.00	1528.00	0
			Proposed	1222.00	1222.00	1222.00	0
ORB10	10×10	944	PSO	1039.00	1263.00	1150.05	48.84
			IGA	1431.00	2121.00	1761.25	158.12
			DE	1190.00	1293.00	1244.40	25.04
			SSO-DM	1114.00	1114.00	1114.00	0
			Proposed	944.00	944.00	944.00	0
YN4	20×20	968	PSO	1340.00	1607.00	1425.15	64.84
			IGA	1826.00	2192.00	1997.90	116.48
			DE	1486.00	1601.00	1570.75	26.15
			SSO-DM	1492.00	1492.00	1492.00	0
			Proposed	979.00	996.00	987.43	6.04

Table 6 shows the experimental results of the proposed algorithm on the instances of Yamada and Nakano [40] (YN1~YN4) and Storer et al. [41] (SWV01~SWV10). In the experiment, the running time limit of the algorithm is set to 2000 seconds (Sec.), and the relative error (RE), i.e., the error between the obtained and the best know solutions defined as a percentage of the best known solution, is introduced as a criterion. The third column BKS/UB in the table represents the best known solutions (BKS) or the known upper bounds (UB) when the BKS is unknown. The last column t shows the running time taken by the algorithm in seconds. The results show that the proposed algorithm can find the best known solutions for the three instances SWV01~SWV03 in a short time, but cannot find the best known solutions for the remaining 11 instances within the running time limit of 2000 seconds. The maximum RE value for these instances do not exceed 6%.

**Table 6:** Simulation test on examples YN01~YN04 and SWV01~SWV10.

Instances	Size	BKS/UB	Best	RE(%)	t (Sec.)
SWV01	20×10	1407	1407	0	1021.06
SWV02	20×10	1475	1475	0	468.86
SWV03	20×10	1398	1398	0	612.78
SWV04	20×10	1474	1505	2.10	2000.00
SWV05	20×10	1424	1506	5.75	2000.00
SWV06	20×15	1678	1746	4.05	2000.00
SWV07	20×15	1600	1630	1.86	2000.00
SWV08	20×15	1763	1798	1.99	2000.00
SWV09	20×15	1661	1724	3.79	2000.00
SWV10	20×15	1767	1795	1.58	2000.00
YN1	20×20	885	896	1.24	2000.00
YN2	20×20	909	912	0.30	2000.00
YN3	20×20	892	905	1.46	2000.00
YN4	20×20	968	979	1.14	2000.00

## 5. Conclusions

Based on the DNA operations of the Adleman-Lipton model, an appropriate encoding strategy is developed first to generate all possible solutions in parallel using DNA computing in one step. Then four highly efficient and parallel DNA algorithms as components of the proposed algorithm are proposed for JSSP. The proposed algorithm is simulated and compared with several heuristics using 58 JSSP benchmark instances from the literature, and the proposed algorithm found the best known solutions for 46 instances. The results show that the proposed algorithm performs better than the comparative heuristics.

One direction of future works is to explore the possibility of solving the FJSP by using viable biological computational models including the sticker model among others. In addition, for larger-scale benchmarks such as the SWVs and the YNs, multi-threaded computing will be considered for simulation implementation in the future.

## Supporting information

S1 File Code, 58 benchmark instances and their data descriptions, solution results and operations guides (Readme.doc file) related to the python source program. (ZIP)
