# Supplementary material for: A DNA algorithm for the job shop scheduling problem based on the Adleman-Lipton model
Source: PLoS One. 2020 Dec 2;15(12):e0242083. doi: 10.1371/journal.pone.0242083 (PMC7710087; doi:10.1371/journal.pone.0242083)
Supplement: S1 File — (ZIP) [file pone.0242083.s001.zip › Python source program/solutions/solution-Example.html]

M1

M2

M3

M4

M5

M6

5

10

15

20

25

30

35

40

45

50

j1t1

j1t2

j1t3

j1t4

j1t5

j1t6

j2t1

j2t2

j2t3

j2t4

j2t5

j2t6

j3t1

j3t2

j3t3

j3t4

j3t5

j3t6

j4t1

j4t2

j4t3

j4t4

j4t5

j4t6

j5t1

j5t2

j5t3

j5t4

j5t5

j5t6

Instance:  Example Size: 5\*6 Makespan: 45
