# Supplementary material for: A DNA algorithm for the job shop scheduling problem based on the Adleman-Lipton model
Source: PLoS One. 2020 Dec 2;15(12):e0242083. doi: 10.1371/journal.pone.0242083 (PMC7710087; doi:10.1371/journal.pone.0242083)
Supplement: S1 File — (ZIP) [file pone.0242083.s001.zip › Python source program/solutions/solution-FT20.html]

M1

M2

M3

M4

M5

50

100

150

200

250

300

350

400

450

500

550

600

650

700

750

800

850

900

950

1000

1050

1100

1150

1200

1250

j1t1

j1t2

j1t3

j1t4

j1t5

j2t1

j2t2

j2t3

j2t4

j2t5

j3t1

j3t2

j3t3

j3t4

j3t5

j4t1

j4t2

j4t3

j4t4

j4t5

j5t1

j5t2

j5t3

j5t4

j5t5

j6t1

j6t2

j6t3

j6t4

j6t5

j7t1

j7t2

j7t3

j7t4

j7t5

j8t1

j8t2

j8t3

j8t4

j8t5

j9t1

j9t2

j9t3

j9t4

j9t5

j10t1

j10t2

j10t3

j10t4

j10t5

j11t1

j11t2

j11t3

j11t4

j11t5

j12t1

j12t2

j12t3

j12t4

j12t5

j13t1

j13t2

j13t3

j13t4

j13t5

j14t1

j14t2

j14t3

j14t4

j14t5

j15t1

j15t2

j15t3

j15t4

j15t5

j16t1

j16t2

j16t3

j16t4

j16t5

j17t1

j17t2

j17t3

j17t4

j17t5

j18t1

j18t2

j18t3

j18t4

j18t5

j19t1

j19t2

j19t3

j19t4

j19t5

j20t1

j20t2

j20t3

j20t4

j20t5

Instance:  FT20 Size: 20\*5 Makespan: 1165
