# Supplementary material for: A DNA algorithm for the job shop scheduling problem based on the Adleman-Lipton model
Source: PLoS One. 2020 Dec 2;15(12):e0242083. doi: 10.1371/journal.pone.0242083 (PMC7710087; doi:10.1371/journal.pone.0242083)
Supplement: S1 File — (ZIP) [file pone.0242083.s001.zip › Python source program/solutions/solution-LA20.html]

M1

M2

M3

M4

M5

M6

M7

M8

M9

M10

50

100

150

200

250

300

350

400

450

500

550

600

650

700

750

800

850

900

950

1000

j1t1

j1t2

j1t3

j1t4

j1t5

j1t6

j1t7

j1t8

j1t9

j1t10

j2t1

j2t2

j2t3

j2t4

j2t5

j2t6

j2t7

j2t8

j2t9

j2t10

j3t1

j3t2

j3t3

j3t4

j3t5

j3t6

j3t7

j3t8

j3t9

j3t10

j4t1

j4t2

j4t3

j4t4

j4t5

j4t6

j4t7

j4t8

j4t9

j4t10

j5t1

j5t2

j5t3

j5t4

j5t5

j5t6

j5t7

j5t8

j5t9

j5t10

j6t1

j6t2

j6t3

j6t4

j6t5

j6t6

j6t7

j6t8

j6t9

j6t10

j7t1

j7t2

j7t3

j7t4

j7t5

j7t6

j7t7

j7t8

j7t9

j7t10

j8t1

j8t2

j8t3

j8t4

j8t5

j8t6

j8t7

j8t8

j8t9

j8t10

j9t1

j9t2

j9t3

j9t4

j9t5

j9t6

j9t7

j9t8

j9t9

j9t10

j10t1

j10t2

j10t3

j10t4

j10t5

j10t6

j10t7

j10t8

j10t9

j10t10

Instance:  LA20 Size: 10\*10 Makespan: 902
