# Supplementary material for: A DNA algorithm for the job shop scheduling problem based on the Adleman-Lipton model
Source: PLoS One. 2020 Dec 2;15(12):e0242083. doi: 10.1371/journal.pone.0242083 (PMC7710087; doi:10.1371/journal.pone.0242083)
Supplement: S1 File — (ZIP) [file pone.0242083.s001.zip › Python source program/solutions/solution-LA36.html]

M1

M2

M3

M4

M5

M6

M7

M8

M9

M10

M11

M12

M13

M14

M15

50

100

150

200

250

300

350

400

450

500

550

600

650

700

750

800

850

900

950

1000

1050

1100

1150

1200

1250

1300

1350

j1t1

j1t2

j1t3

j1t4

j1t5

j1t6

j1t7

j1t8

j1t9

j1t10

j1t11

j1t12

j1t13

j1t14

j1t15

j2t1

j2t2

j2t3

j2t4

j2t5

j2t6

j2t7

j2t8

j2t9

j2t10

j2t11

j2t12

j2t13

j2t14

j2t15

j3t1

j3t2

j3t3

j3t4

j3t5

j3t6

j3t7

j3t8

j3t9

j3t10

j3t11

j3t12

j3t13

j3t14

j3t15

j4t1

j4t2

j4t3

j4t4

j4t5

j4t6

j4t7

j4t8

j4t9

j4t10

j4t11

j4t12

j4t13

j4t14

j4t15

j5t1

j5t2

j5t3

j5t4

j5t5

j5t6

j5t7

j5t8

j5t9

j5t10

j5t11

j5t12

j5t13

j5t14

j5t15

j6t1

j6t2

j6t3

j6t4

j6t5

j6t6

j6t7

j6t8

j6t9

j6t10

j6t11

j6t12

j6t13

j6t14

j6t15

j7t1

j7t2

j7t3

j7t4

j7t5

j7t6

j7t7

j7t8

j7t9

j7t10

j7t11

j7t12

j7t13

j7t14

j7t15

j8t1

j8t2

j8t3

j8t4

j8t5

j8t6

j8t7

j8t8

j8t9

j8t10

j8t11

j8t12

j8t13

j8t14

j8t15

j9t1

j9t2

j9t3

j9t4

j9t5

j9t6

j9t7

j9t8

j9t9

j9t10

j9t11

j9t12

j9t13

j9t14

j9t15

j10t1

j10t2

j10t3

j10t4

j10t5

j10t6

j10t7

j10t8

j10t9

j10t10

j10t11

j10t12

j10t13

j10t14

j10t15

j11t1

j11t2

j11t3

j11t4

j11t5

j11t6

j11t7

j11t8

j11t9

j11t10

j11t11

j11t12

j11t13

j11t14

j11t15

j12t1

j12t2

j12t3

j12t4

j12t5

j12t6

j12t7

j12t8

j12t9

j12t10

j12t11

j12t12

j12t13

j12t14

j12t15

j13t1

j13t2

j13t3

j13t4

j13t5

j13t6

j13t7

j13t8

j13t9

j13t10

j13t11

j13t12

j13t13

j13t14

j13t15

j14t1

j14t2

j14t3

j14t4

j14t5

j14t6

j14t7

j14t8

j14t9

j14t10

j14t11

j14t12

j14t13

j14t14

j14t15

j15t1

j15t2

j15t3

j15t4

j15t5

j15t6

j15t7

j15t8

j15t9

j15t10

j15t11

j15t12

j15t13

j15t14

j15t15

Instance:  LA36 Size: 15\*15 Makespan: 1268
