# Supplementary material for: A DNA algorithm for the job shop scheduling problem based on the Adleman-Lipton model
Source: PLoS One. 2020 Dec 2;15(12):e0242083. doi: 10.1371/journal.pone.0242083 (PMC7710087; doi:10.1371/journal.pone.0242083)
Supplement: S1 File — (ZIP) [file pone.0242083.s001.zip › Python source program/Readme.doc]

1. The Python version used is Python 3.6.10, and the environment configuration is based on Anaconda, as shown in the following figure, the environment is named "python36", and this environment name will be called on the command line.

2. Since the "cplex" solver needs to be called when using "docplex" in Python, you need to install IBM Cplex Studio in advance before installing the "docplex" library. We are using the academic version of CPLEX Studio IDE 12.10.0, which can be downloaded from the IBM official website.

(https://www.ibm.com/analytics/cplex-optimizer)

3. Under the installation path of CPLEX Studio IDE 12.10.0 (for example, our installation path is:...\program\cplex\python\3.6\x64_win64), find the "cplex" folder, as follows:

4. Copy the "cplex" folder as a whole to the "site-packages" folder of the above python36 environment directory (for example, our path is:

E:\Anaconda\Anaconda2019\envs\python36\Lib\site-packages).

5. Use the commands "pip install docplex" and "pip install biopython" in the command line to install the "docplex" library and the "Biopython" library. Note that you must install it in the environment named "python36" mentioned above. Or, you can perform visual installation in Anaconda environment as follows. The installation of other package libraries used in the program can refer to the above two schemes.

6. Now you can test the program. Enter "activate python36" on the command line to enter the "python36" environment:

7. Change the path to "...\Python source program\code\JSSP" as shown below:

8. Enter the run command "python JSSP.py ft20". Among them, JSSP.py is the main program, and ft20 is the test data, which can be replaced with other test examples provided in this paper.

9. If the following result is displayed, it means the program runs successfully. You can view the running results in the "solutions" folder:

10. It should be noted that because the Makespan value of the test instance is generally large, the time grid interval of the Gantt chart is set to 50. We have edited Example 3 in this paper as a test instance. If you want to test and verify Example 3, please adjust the parameter "solution.visualize (time_factor = 1, time_grid = 50)" in line 50 of the main program "JSSP.py" to "time_factor = 15, time_grid = 5", and then execute the "python JSSP.py Example" command on the command line.

(time_factor = 1, time_grid = 50)	(time_factor = 15, time_grid = 5)
